# Presentation of a new multifunctional oral cavity simulator: the “MOCS”

**DOI:** 10.1590/1807-3107bor-2025.vol39.022

**Published:** 2025-02-21

**Authors:** Tamires Timm MASKE, Maximiliano Sérgio CENCI, Rafael PATZLAFF, Iniative MOCS, Lina Naomi HASHIZUME, Marisa MALTZ, Rodrigo Alex ARTHUR

**Affiliations:** (a) Universidade Federal do Rio Grande do Sul – UFRS, Department of Preventive and Community Dentistry, Porto Alegre, RS, Brazil.; (b) Radboud University Medical Center, Department of Dentistry, Nijmegen, The Netherlands.; (c) Odeme Dental Research, Luzerna, SC, Brazil.; (d) O Universidade Federal do Rio Grande do Sul, Department of Preventive and Community Dentistry, Federal University of Rio Grande do Sul, Porto Alegre, RS, Brazil.

**Keywords:** Tooth Abrasion, Erosion, Dental caries, Demineralization, Biofilm, Dental Materials

## Abstract

This article describes a new multifunctional oral cavity simulator (MOCS) that allows, with little technical support and easy handling, the laboratory development of dental hard tissue lesions under clinically similar conditions. The MOCS consists of a heating unit containing three independent cylindrical chambers with three specimen holders inside. Liquids flow through the surfaces of specimens by inlets on the lid of the chamber, which is connected to a medium/artificial saliva source through a computer-controlled peristaltic pump. The design, operational principles, and clinical application of this simulator, such as microcosm-induced development of carious-like lesions, acidic-induced erosion-abrasion like-lesions, and testing the anticariogenic effect of restorative materials, are shown. The MOCS can mimic several aspects of the oral cavity, being a promising device for assessing dental hard tissue lesions.

## Introduction

The human oral cavity is a complex environment where teeth are subjected to various challenges, including mechanical, chemical, and thermal ones, potentially affecting the integrity of dental tissues. In this respect, clinical studies are the most relevant for daily clinical practice because they take into account this complexity. However, the time needed to assess clinically relevant outcomes and participant adherence are some of the shortcomings of clinical studies. A detailed assessment of dental tissue’s chemical, mechanical, and structural characteristics is impossible unless the tooth is scheduled to be extracted and subsequently analyzed by destructive methods. Therefore, developing reliable, validated, low-cost, and user-friendly laboratory models has been encouraged as first-level evidence to guide the design of further clinical studies.^1,2^In laboratory models, the outcomes can be assessed relatively quickly and with simpler logistics considering workforce, participant recruitment, and compliance issues.^1^ Therefore, there is a vast literature on the use of laboratory models for the study of dental tissue pathologies, such as dental caries and dental erosion, whose data have helped to delineate strategies for the prevention, control, and treatment of these conditions.

Dental caries and dental erosion are pathologies characterized by the loss of mineral content in dental tissues but presenting distinct etiological factors. While the former is induced by organic acids produced by microbial biofilm metabolism in response to dietary sugars, the latter is caused by acids that do not originate from microorganisms.^2,4^ Yet, the interplay between the exposure of tooth surfaces to non-microbial acids and mechanical forces (e.g. tooth brushing or tongue movements) accelerates mineral loss due to a chemical-mechanical process (erosive and abrasive), resulting in a complex condition known as erosive tooth wear (ETW).^2^ Several in vitro models, varying from the simplest to highly complex assemblies, are available to mimic the development of both carious and erosive lesions (associated or not with mechanical wear)^3,8^. Among them, dynamic Artificial Mouth Systems (AMS) stand out as complex models, as they mimic important factors of the oral cavity, such as salivary flow, dietary-mediated pH oscillations, temperature, and ideal atmosphere for biofilm growth. AMS may provide new insights into caries prevention and control strategies by evaluating distinct dietary compounds and anticariogenic or antimicrobial substances. They also allow the evaluation of restorative materials, considering their anticariogenic properties and the carious lesions development around restorations under clinically relevant conditions. Furthermore, AMS enables the study of ETW-associated factors, such as the type and concentration of acids and the role played by other factors that could potentially protect dental surfaces or affect the extent of dental wear^4^.

With AMS, many variables can be assessed simultaneously, a large number of specimens can be tested, and the variation between specimens can be analyzed without the confounding variables of the subject and the oral environment ^1^. Nevertheless, these models require a sophisticated and intricate assembly, often posing challenges in handling, and not fitting well to the laboratory infrastructure. Therefore, this study aimed to describe and prove the operational principles of an efficiently laboratory model for the study of dental mineral loss. This new artificial mouth system is an improved and updated version of the apparatus proposed by Maske et al. (2016) ^5^ with some assembling differences. The simulator, coined as Multifunctional Oral Cavity Simulator (MOCS), was designed to allow the development of the biofilm microcosm and carious lesions and simulate aspects related to erosive-abrasive lesion development. The hypothesis tested in this investigation was that the MOCS can be used to develop carious and erosive-abrasive lesions under clinically relevant conditions.

## Methods

### Ethical aspects

This study protocol was reviewed and approved by the local Research Ethics Committee (School of Dentistry, Federal University of Rio Grande do Sul, Porto Alegre, RS, Brazil) under protocol number CAAE 43894821.7.0000.5347. Written informed consent was obtained from all participants in this study.

### Design and operational principle

**Figure 1:**
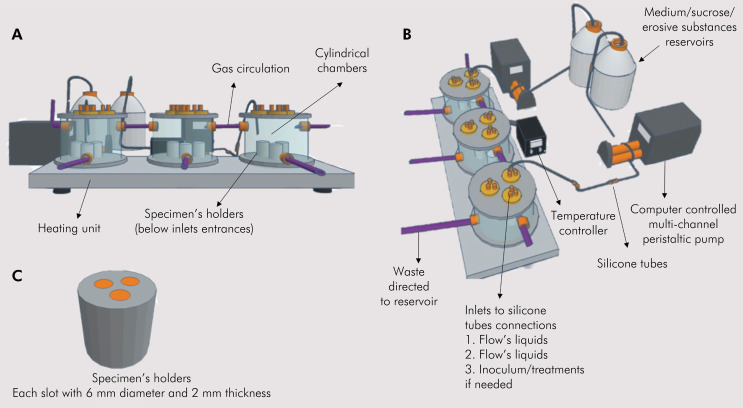
A) MOCS front view showing the heating unit with cylindrical chambers and gas circulation into the system. B) Lateral view showing the inlets for introducing liquids, inoculum, or treatment, the computer-controlled peristaltic pumps connected to silicone tubes from reservoirs, and the connections to the waste reservoir. C) Holder for specimen accommodation.

The MOCS consists of a heating unit containing three independent cylindrical chambers with three specimen holders inside. Liquids released by inlets located on the lid of the chamber flow through the surfaces of specimens. These inlets are connected to a medium/artificial saliva source by a computer-controlled peristaltic pump (Figure 1A and 1B). Each holder lodges up to 3 different specimens (Figure 1C). The cylindrical chambers are maintained at a constant and controlled temperature (37^o^ C) by a heating unit by conduction. Each chamber is built with upper and lower stainless-steel covers and a cylinder of polypropylene plastic (which is autoclavable). There are two inlets in this cylinder: one at the top of the cylinder, through which the anaerobic gas flows through the three chambers and the other in the bottom part of that cylinder, through which liquids can be removed from the system (Figure 1A).

The waste produced by the liquids is directed to an external reservoir. Gas (10% CO_2_, 10% H_2_, and a balance of 80% N_2_) circulates through the chambers twice daily (every 12 hours for 1 minute, 1 Psi / 0.007N/mm^2^).

The top cover of the cylinder has nine inlets for the entrance of liquids to the system. One can be used for inserting artificial saliva, another for diet exposure (sucrose, erosive substance), and the others for tested liquids, such as microbial inoculum or liquid/slurry treatments (Figure 1A). The inlets are connected to the peristaltic pump by silicone tubes leading to the liquid reservoirs. The pumps are controlled by computer, so that different frequencies and flow rates can be simulated (Figure 1B).

The MOCS was designed to allow liquids to be perfused into the system in different frequencies and flowrates, either mimicking salivary flow (which is simulated by artificial saliva or culture medium flow) and a cariogenic diet (different sucrose frequencies), enabling biofilm formation by microbial inoculum, or mimicking erosive wear by an erosive diet (erosive beverages, for example).

### Clinical-like applications of the MOCS

#### Example 1. Carious-like lesion development

**Figure 2:**
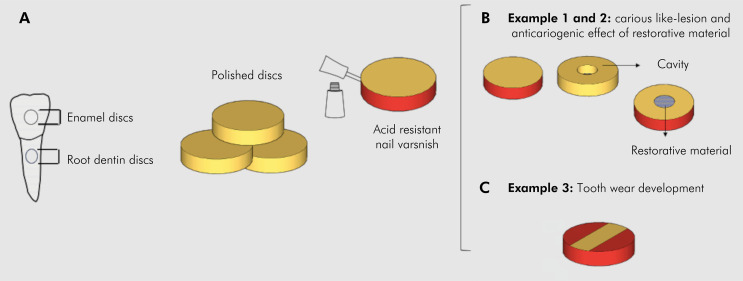
Specimen preparation, for example 1 – carious like-lesion, example 2 – anticariogenic effect of restorative material, and example 3 – tooth wear development. A) Bovine tooth and polished discs prepared from them and application of acid-resistant nail varnish. B) Dentin/enamel discs for carious like-lesion development and enamel discs with a cavity prepared to receive anticariogenic or placebo restorative material. C) Dentin/enamel discs prepared for tooth wear development.

Dental hard tissues were obtained from sound bovine teeth. Enamel and dentin specimens were prepared as discs (6 mm diameter and 1.7 mm thickness) from a trephine drill (8 mm diameter). Specimens were polished with sandpaper (#200, #600, #1200, and #2000) under water refrigeration (Figure 2A). The disc specimens were protected with acid-resistant varnish, except dentin or enamel surfaces, which had their hardness assessed by three indentations 50 µm apart on the surface center made using Knoop diamond indenter loaded with 50 and 25 g weights for 5 s for enamel and dentin specimens, respectively. The surface hardness analysis was assessed before (baseline) and after the biofilm formation using a Micro Hardness Tester (FM 700, Future-Tech Corp., Tokyo, Japan)^5^.

Saliva was used as microbial inoculum to allow microcosm biofilm growth on enamel and dentin specimens previously sterilized with ethylene oxide (M.I.C. Esteriliza, Porto Alegre, RS, Brazil). The inoculum (5 mL) was collected from healthy subjects (without systemic diseases and oral diseases, such as dental caries and periodontal diseases) who abstained from food ingestion for 2 hours before the collection and did not use antibiotics in the last 3 months. Microcosm biofilms formed over enamel and dentin specimens were grown from saliva donated by one male subject (24 years old) and one female subject (35 years old).

The saliva was individually collected and mixed with 100 mL of artificial saliva (Defined Medium Mucin; DMM.)^6^ (Sissons and Wong, 2001). The mixture was kept on the sterilized enamel or dentin surfaces for 1 h. Subsequently, the artificial saliva (D.M.M.) was perfused at a 0.06 mL/min flowrate with intermittent 5% sucrose regimen (3x/ day for 6 min at 0.25 mL/min flowrate) according to Maske et al. (2016) ^5^. The microcosm biofilms were grown undisrupted for up to 7 days (4 and 7 days for dentin and 7 days for enamel)^5^. The biofilm formed on the tested samples was collected after 4 and 7 days of biofilm growth, weighted, added to 1 mL of saline solution, sonicated (20W, 30 s), serially diluted, and plated in duplicat (20 µL) in Brain Heart Infusion Agar (BHI) and Mitis Salivarius Agar (MSB) supplemented with 0.2 U bacitracin per mL to allow the cultivation of total and mutans group viable microorganisms, respectively. The agar plates were stored at 37ºC in anaerobiosis jars for 4 days. Microorganism counts were expressed as colony forming units (CFU) per milligrams of biofilm collected. The primary outcome was carious lesion development evaluated by enamel/dentin surface hardness assessment and the secondary outcome was viable cell count. As all specimens had their surface hardness determined at baseline, each enamel/dentin specimen was its own control. In each experimental time point, the paired-T test was used to analyze enamel and dentin demineralization data (baseline and after biofilm formation).

#### Example 2. Anticariogenic effect of restorative material

To test the anticariogenic effect of a restorative material, cylindrical slots of 1.8 mm diameter were prepared in the center of enamel discs (Figure 2B)^7^. Dental restorations were prepared using a pre-reacted glass ionomer that releases fluoride, phosphate, and carbonate ions (S-PRG Barrier Coat, Shofu, Japan) according to the manufacturer’s instructions and placed into the slots (Figure 2B). Controls consisted of a modified S-PRG without ions release. The samples were sterilized with ethylene oxide. Microcosm biofilms were formed onto those discs for 7 days by saliva inoculum provided by a healthy donor (24 years old) following the protocol described above. Both the baseline and final superficial hardness were evaluated around the restoration. An average of three indentations spaced 100 µm from the restoration and 50 µm from each other were made at baseline (SH1) and after the biofilm formation (SH2). A percent surface hardness change was calculated as follows: %SHC =100 (SH2-SH1)/SH1^8^.

#### Example 3. Tooth wear development through an erosive-abrasive model

Tooth wear on enamel and dentin specimens was induced by an erosive-abrasive model mimicking an extrinsic acid source. Enamel and dentin specimens were prepared from sound bovine teeth using a trephine drill to obtain discs (3 mm diameter and 1.5 mm thickness). Specimens were polished with sandpapers with decreasing granulation (#400, #800, #1200, and #4000) under water refrigeration (Arotec, APL-04 model, Cotia-SP, Brazil). An optical profilometer was used to evaluate the discs’ surface (Bruker, ContourGT-K, Berlin, Germany). The curvature of specimens was determined using Vision64TM software (Bruker, ContourGT-K, Berlin, Germany), and only those showing mean variations of up to 0.3 µm were selected ^9^. Disc surfaces were covered by acid-resistant nail varnish, leaving a rectangular window uncovered at the center of the specimens (1 mm wide x 3 mm long) to be exposed to the test solutions (Figure 2C).

A soft drink with high erosive potential (Coca-Cola, Ind. Brasileira de Bebidas S.A. Jundiaí – S.P., Brazil) was used as an extrinsic acid source. Artificial saliva^10^ was perfused into the system and dropped into the specimens’ surface at a flow rate of 0.2 mL/min during the day and at a flow rate of 0.04 mL/min during the night (8 h) to simulate the circadian cicle of saliva (higher during the day and lower at night). During the day, distilled water (control) and the soft drink (test) were perfused into the system 3 times per day (every 4 hours) for 10 minutes (0.25 mL/min). This frequency of exposure was chosen based on an in situ study proposed by Honório et al. (2010) ^11^. To simulate mechanical abrasion on the dental tissues, the holders were removed from the system, and the specimens were brushed for 10 seconds each with a slurry of conventional fluoridated dentifrice (1/3; 1,100 ppm F, NaF, Sorriso, Colgate-Palmolive, São Paulo-SP, Brazil) using a toothbrush with soft bristles in standardized brushing machine with 2N^9^ force and 60 rpm before the first and after the last erosive challenge of the day. According to ADA recommendation^12^, around 2 minutes is needed to brush all teeth (32 in total), which means that approximately 10 seconds are needed to clean the 3 samples in the holder. After 5 days of erosion-abrasion cycling, the optical profilometry was again used on specimens to determine the step height (representing the tooth mineral wear) formed after the erosive/abrasive challenge. After removing the nail varnish, the unexposed tooth surfaces were considered as reference for step height assessment. Each specimen was assessed three times (across unexposed and exposed surfaces), and surface loss readings were averaged using the Vision64TM surface metrology software.

## Results

For the carious-like lesion development, the enamel and dentin surface hardness after microcosm biofilm formation was about three times lower compared with baseline hardness values, corresponding to a 60 to 70% decrease in surface hardness for enamel and dentin samples (Table 1). Table 2 shows the microbiological data for distinct biofilm growth and tooth substrate. Total microorganisms and mutans streptococci were found in all tested conditions showing that a viable biofilm was cultivated over the 7 days.

**Table 1:** Superficial hardness values (mean ± sd) from sound enamel or dentin discs (baseline) and after biofilm formation in the MOCS.

Experimental days	Enamel carious-like lesion		Dentin carious-like lesion	
	Hardness kgF/mm^2^		Hardness kgF/mm^2^	
	Baseline	After biofilm formation	Baseline	After biofilm formation
4 days (n=9)	-	-	48.46±5.17^A^	13.96±8.35^B^
7 days (n=12)	316.67 ±19.28^A^	106.42± 86.46^B^	49.81±6.84^A^	15.59±9.27^B^

Note: In each dental substrate, different upper-case letters represent statistically significant differences between hardness measurements before and after biofilm formation in the MOCS system (p<0.01, Paired T-test). A decrease in hardness values (baseline versus after biofilm formation) indicate tooth mineral loss.

**Table 2:** Microbial counts of biofilms (mean ± sd; CFU x 106/mg biofilm) formed during the tested experimental times.

	Enamel (n=12) *	Dentin (n=9) **	
	7 days	4 days	7 days
Total microorganisms	1.83±2.24	6.19 ±5.05	7.94 ± 7.80
Mutans streptococci	4.35±14.96	0.88±0.57	1.11 ±0.80

*Saliva inoculum from a male donor (24 years old).

** Saliva inoculum from a female donor (35 years old).

For the anti-cariogenic restorative material test, the percent surface hardness change was 66.3% and 32.17% around placebo and around anti-cariogenic pre-reacted glass ionomer restorations, respectively, being the difference between groups statistically significant (T-test, p<0.05).

**Figure 3:**
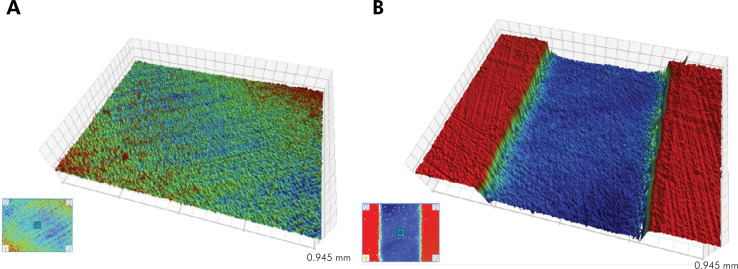
Representative optical profilometer analysis of an enamel sample exposed to distilled water (A) and soft drink (B): 3D surface topography of profilometric sample measurements. In (A), there is no difference in depths. In (B), the red zone represents the unexposed tooth surface (previously protected by acid-resistant nail varnish) and the deep blue area represents the surface exposed to the soft drink.

For the erosive-abrasive-like lesions, the step height data for each dental substrate were compared between soft drinks and distilled water by t-test and linear regression analysis. Table 3 and Figure 3 show that after 5 days of cycling, a greater step height was found on specimens exposed to the tested soft drink. There was a significative effect on step height for soft drink in dentin (8.59 µm; CI 8.42-8.75; p< 0.001, adj R^2^ =0.99) and enamel (9.55 µm; CI 9.22-9.87; p<0.001; Adj R^2^ =0.99).

**Table 3:** Step height (mean ± sd; µm) of enamel and dentin specimens according to exposure to soft drink or distilled water.

	Soft drink (Coca-cola®)	Distilled water (control)
Enamel (n=9)	9.85 (0.46) ^A^	0.30(0.03) ^B^
Dentin (n=9)	8.87 (0.24) ^A^	0.28 (0.04) ^B^

Note: In each substrate, different upper-case letters represent statistically significant differences between the tested groups (T-test, p<0.05).

## Discussion

The examples provided in this study support the versatility of the Multifunctional Oral Cavity Simulator. The MOCS produced enamel and dentin carious-like lesions, confirmed by the decrease in surface hardness at 4 and 7 days after microcosm biofilm formation. The decrease in enamel hardness was similar to the chemically-induced demineralization (around 70%) in pH-cycling models on human enamel over 10 days of cariogenic challenge^13^. Compared with other microbial biofilm models, the extent of demineralization was similar to the one induced by a static microbial consortium on bovine enamel under continuous exposure to sucrose and fluoride for 4 days^14^. Moreover, the demineralization found in the present model was slightly more aggressive than that of a similar dynamic microcosm biofilm model that showed 50-60% demineralization after 7 days of experiment using the same protocol for enamel demineralization^5^. Concerning the dentin substrate, our findings were similar to that of static dual-species biofilm and continuous exposure to sucrose^15^. Static microcosm biofilm models using bovine dentin as substrate have shown approximately 56-67% of mineral loss when 20% sucrose was applied 3 or 6 times daily^16^. The same level of demineralization was also observed on human enamel and on bovine dentin by pre-clinical in situ models during 14 and 7 days, respectively^17,18^. Although the models differ on the extent of induced demineralization, which could be related to the differences in design and experimental conditions, the MOCS was able to create early carious lesions, which opens possibilities for further testing preventive of therapeutic strategies.

Similarly to the dynamic microcosm biofilm model proposed by Maske et al. (2016), in example 1, the mutans streptococci counts were higher due to the selective pressure by exposure to sucrose, indicating that the proposed MOCS simulates the microbial shifts proposed by the plaque ecological theory ^19^. Data from example 1 also indicated that the microcosm biofilms formed under dietary carbohydrate source (sucrose) were able of causing dental mineral loss similar to a clinical situation where a patient with a cariogenic diet and poor oral hygiene develops carious lesions^20^. In this study, surface hardness was used instead of cross-sectional hardness and transversal microradiography (TMR) (the gold-standard method for assessing dental tissue lesion development/arrestment^21^) to assess subsurface lesions. Previous studies have shown a good correlation between surface hardness assessment and TMR for both enamel and dentin lesions^14,18,22^. Besides, surface hardness can be considered as a calibrating measure for lesion preparation, which was the case in this study. Further investigations considering cross-sectional examinations and even x-ray light-based methods will be planned to evaluate physical and chemical changes in the lesions formed by this simulator.

In example 2, the anticariogenic effect of a dental material was demonstrated. The pre-reacted glass ionomer S-PRG used is considered a biomaterial with tooth mineralization proprieties. Many studies have illustrated that the release of ions, including fluoride and others, from the S-PRG filler are absorbed by adjacent enamel and dentin, preventing demineralization and protecting the tooth surface from acid-induced demineralization^24,25^. Consistent with these findings, the MOCS reproduced the caries prevention effect proposed by this dental material, which showed an anticariogenic effect, serving as a valuable tool for future insights into caries prevention strategies.

In example 3, according to the linear regression analysis, the dentin and enamel surface step height increased around 9 µm after five days in the simulator system, indicating that MOCS was able to simulate dentin and enamel erosive wear. Eisenburger et al. (2003) ^26^ alternated erosion and brushing cycles with 2N force in an in vitro study and found approximately 10 µm of enamel wear after citric acid exposure for 40 min. Levy et al. (2011)^27^ used brushing with free-fluoride dentifrice (1.5 N, 10s, 2x/ day) and exposure to Coke 4x/ day and found 3.01 µm of enamel wear. Attin et al. (1998) ^28^ showed approximately 12 µm of dentin wear after soft drink exposure and brushing (200/min, 2.75 N). In an in situ study, Magalhães et al. (2008) ^29^ demonstrated that Coke (4x/ day for 1 min) associated to brushing for 10 s worn approximately 5 µm of dentin surface. Hooper et al. (2003) ^30^ used orange juice associated to fluoride dentifrice and demonstrated a 1-2-µm wear in enamel and a 41-49-µm wear in dentin substrates. Meaningful comparisons between erosion-abrasion investigations are difficult due to different methodological protocols and parameters between studies. However, erosion-abrasion lesions were created in the MOCS with similar values from findings in the literature and therefore, this simulator can be reliably indicated for further investigation in ETW studies.

Artificial mouth systems have been used to study dental erosion^1,29,31^, but the role of abrasion was not considered in the available apparatus. Besides reproducing the circadian oscillations of salivary flow and the daily erosive challenges that the oral cavity is exposed to, the MOCS system incorporated the daily toothbrushing cycle to further simulate a clinical condition. The specimens exposed to distilled water (control group) did not show visible step height change: only a slight alteration on the exposed surface (step height ~0.3 µm) was observed when to the sound polished tooth surface^32^. It is known that erosive challenges soften the tooth surface facilitating the mechanical removal of dental tissues and the occurrence of ETW^33,34^.

Besides the advantages in the ETW studies, the MOCS system was also able to simulate important clinical factors for carious lesion development. The inoculum used in this simulator was provide by saliva, which is a microcosm from the oral cavity ecosystem^35^. This kind of inoculum has the advantage of maintaining the heterogeneity and complexity of the original sample and allows microbial community dynamics to be replicated within a laboratory environment^36^. Therefore, the dental demineralization in this model is the result of the global metabolic activity of the selected ecosystem, rather than the specific activity of a single species or consortium-defined inoculum. This improves the similarity between the model and the carious lesions development in the oral cavity. Moreover, the MOCS allows the formation of biofilm under a dynamic culture, better reproducing the characteristics of the oral cavity such as salivary and nutritional flow and the shear forces acting in the oral biofilm formation. Therefore, the biofilm grows with a continuous or intermittent nutrient flow, reproducing the cycles of scarcity and abundance of nutrients and the removal of metabolic nutrients, such as in the oral cavity ^37,38^.

It must be acknowledged that in situ models of the mouth, in which dental lesions can develop in real-world clinical conditions, have a higher complexity compared to the MOCS. However, complex models such as the MOCS system are useful because they can be executed in a shorter period of time, require fewer staff than in situ studies, have no problems with participant compliance and are relatively cheaper than in situ studies^1^.Therefore, models designed to mimic the oral cavity, such as the MOCS system, that provide data on caries lesions and erosive-abrasion that correlate with in situ results are valuable for preliminary work before embarking on more expensive clinical studies.

The data presented in this study show promising applications of the MOCS device. We have first shown the application of this model for producing carious and erosive-abrasive like-lesions as well as for evaluating the anticariogenic effect of a restorative material with comparable results to those obtained under clinically similar conditions; therefore, the hypothesis of this investigation was confirmed. Future studies should be carried out to test its ability to evaluate dose-response to other anti-cariogenic products (e.g., fluoride, chlorhexidine) and anti-erosive substances.

## Conclusion

The Multifunctional Oral Cavity Simulator (MOCS) was able to mimic several aspects of the oral cavity, being a promising user-friendly device for the assessment of hard dental tissue lesion development, such as dental carious-like and erosive-abrasive-like lesions, requiring little technical support.
